# Postprandial effects of macronutrient composition meals on the metabolic responses and arterial stiffness indices of lean and obese male adults: a protocol of a pilot study

**DOI:** 10.1186/s40814-021-00787-2

**Published:** 2021-02-03

**Authors:** Safieh Firouzi, Reza Rezvani, Naseh Pahlavani, Lida Jarahi, Jamshid Gholizadeh Navashenaq, Golnaz Ranjbar, Mahsa Malekahmadi, Zhila Taherzadeh, Mohammad Safarian

**Affiliations:** 1 Student Research Committee, Mashhad University of Medical Sciences, Mashhad, Iran; 2 Department of Nutrition, Faculty of Medicine, Mashhad University of Medical Sciences, Mashhad, Iran; 3 Social Development and Health Promotion Research Center, Gonabad University of Medical Sciences, Gonabad, Iran; 4 Cellular and Molecular Research Center, Research Institute for Health Development, Kurdistan University of Medical Sciences, Sanandaj, Iran; 5 Department of Community Medicine, School of Medicine, Mashhad University of Medical Sciences, Mashhad, Iran; 6 Noncommunicable Diseases Research Center, Bam University of Medical Sciences, Bam, Iran; 7 Bam University of Medical Sciences, Bam, Iran; 8 Target Drug Delivery Research Center, Mashhad University of Medical Sciences, Mashhad, Iran

**Keywords:** Metabolic responses, Macronutrient composition, Arterial stiffness, Protocol study

## Abstract

**Background:**

Prior studies have shown that meal composition may affect the metabolic responses and arterial stiffness indices, and these responses may be different in lean and obese adults. The primary objective of this study is to determine the feasibility of conducting a trial to compare the effect of three test meals in lean and obese men. Due to the lack of a comprehensive study that concurrently compares metabolic responses and vascular stiffness indices after receiving three different meals in lean and obese men, this pilot study will be conducted with a three-phase parallel design, aiming to investigate the effects of meal composition on the metabolic parameters and arterial stiffness indices of lean and obese adults.

**Methods:**

This pilot, a parallel clinical trial will be performed on 24 male adults aged 18–35 years since January 2021 and will continue until March 2021 who are disease-free and selected based on the inclusion and exclusion criteria at Mashhad University of Medical Sciences, Iran. The subjects will complete three interventions at a 1-week interval, including high carbohydrate (70% carbohydrates, 10% protein, 20% fat), high protein (30% protein, 50% carbohydrates, 20% fat), and high-fat meal (50% fat, 40% carbohydrates, 10% protein). Postprandial effects will be assessed within 360 min after each meal, including the energy expenditure component (resting energy expenditure, thermic effects of feeding, respiratory quotient, and substrate oxidation) and arterial stiffness indices (augmentation index and pulse wave velocity). In addition, blood sampling will be performed to measure glucose, insulin, free fatty acids, and lipid profile.

**Discussion:**

The differences in the postprandial responses can affect the metabolic and vascular parameters due to different meal compositions, thereby providing beneficial data for the establishment of new strategies in terms of nutritional education and metabolic/vascular improvement. Also, the results from this pilot study will inform intervention refinement and efficacy testing of the intervention in a larger randomized controlled trial.

**Trial registration:**

Iranian Registry of Clinical Trials; code: IRCT20190818044552N1; registered on August 26, 2019

## Background

Obesity is a multifactorial, complex, and preventable disorder, which is predicted to affect 20% of the world’s adult population by 2030, while 38% of this population is predicted to become overweight. Epidemiological studies have demonstrated that obesity and overweight are important risk factors for cancer, diabetes, premature death, and cardiovascular diseases [1]. Obesity could be caused by genetic factors, high-energy intake, low-energy expenditure, reduced physical activity, low sympathetic activity, decreased fat oxidation, and differences in hormonal responses [2]. Genetic factors play a pivotal role in the prediction of obesity, and the rising trend of obesity has also highlighted the importance of environmental factors in this regard [2]. The leading causes of obesity include increased energy intake (especially from energy-dense and high-fat foods) and reduced physical activity [3].

Dietary macronutrient composition remarkably influences body weight adjustment. In response to acute changes in the dietary macronutrient composition (e.g., increased fat intake), human subjects have reported the increased oxidation of carbohydrates and total energy expenditure (TEE). Moreover, the short-term changes in the energy intake of human subjects have been shown to stimulate hormonal and metabolic alterations [4]. Postprandial TEE and thermic effect of feeding (TEF) are the main objectives in the management of energy balance, with TEF contributing to postprandial TEE under the possible effect of dietary composition [5–7].

According to human studies, the consumption of animal protein in breakfast leads to less significant changes in the plasma levels of glucose and insulin, while it increased TEF [8–10], and reduced food intake during the day [8, 11]. On the other hand, the vascular function is considered to be a major determinant of coronary artery health, and its disruption could increase the risk of cardiovascular diseases (CVDs) [12]. Previous findings have indicated that the vascular function differs between obese and normal adults in the fasting state [13], while the acute effects of dietary macronutrient composition on the vascular function have not been well documented.

Postprandial hyperglycemia [14, 15], and hypertriglyceridemia [16, 17], have emerged as independent cardiovascular risk factors, promoting interest in the cardiovascular effects of acute dietary exposure. In addition, some findings have demonstrated that postprandial triglyceride (TG) levels may be influenced by the type of lipids, while other studies have shown that saturated fatty acids may induce a slight increase in the levels of postprandial TGs compared to unsaturated fatty acids [18, 19]. A research in this regard indicated that high-fat meals could reduce postprandial cardiovascular reactivity [20]. Another study also demonstrated that diets with high trans fatty acids may increase serum-free fatty acids (FFAs), and high-FFA diets may lead to hyperinsulinemia [21].

Arterial stiffness is an independent risk factor for CVDs, and its intensity may increase in the presence of other CVD risk factors [22, 23]. However, data are scarce regarding postprandial arterial stiffness [24–26], particularly in obese individuals. Aortic pulse wave velocity (PWV) is the ‘gold standard’ for the evaluation of aortic stiffness and subclinical organ damage [27]. Despite the significant association between postprandial lipemia and vascular response, as well as vascular function and arterial stiffness, it remains unknown whether postprandial lipemia directly leads to acute detectable responses in arterial stiffness [28, 29]. According to the above content, prior studies have shown that meal composition may affect the metabolic responses and arterial stiffness indices.

Due to the lack of a comprehensive study that concurrently compares metabolic responses and vascular stiffness indices after receiving three different meals in lean and obese men, the primary objective of this study is to determine the feasibility of conducting a trial to compare the effect of three test meals in lean and obese men and also this pilot study will compare the effects of dietary macronutrient distribution on the metabolic responses (energy expenditure components, postprandial lipemia, and glycemia) and arterial stiffness indices (PWV and pulse wave analysis) of normal and obese adults.

## Methods/design

### Research hypothesis and study time

We will hypothesize that dietary macronutrient composition has different effects on the metabolic responses and arterial stiffness indices of normal and obese adults. The study protocol will be approved by the Research Ethics Committee of Mashhad University of Medical Sciences, and the pilot study will be completed. The study has been initiated since January 2021 and will continue until March 2021. In total, 24 participants (12 obese men and 12 lean men) will complete the interventions and assessments. Data analysis and reporting of the results will be performed until summer of 2021.

### Experimental design

**Fig. 1:**
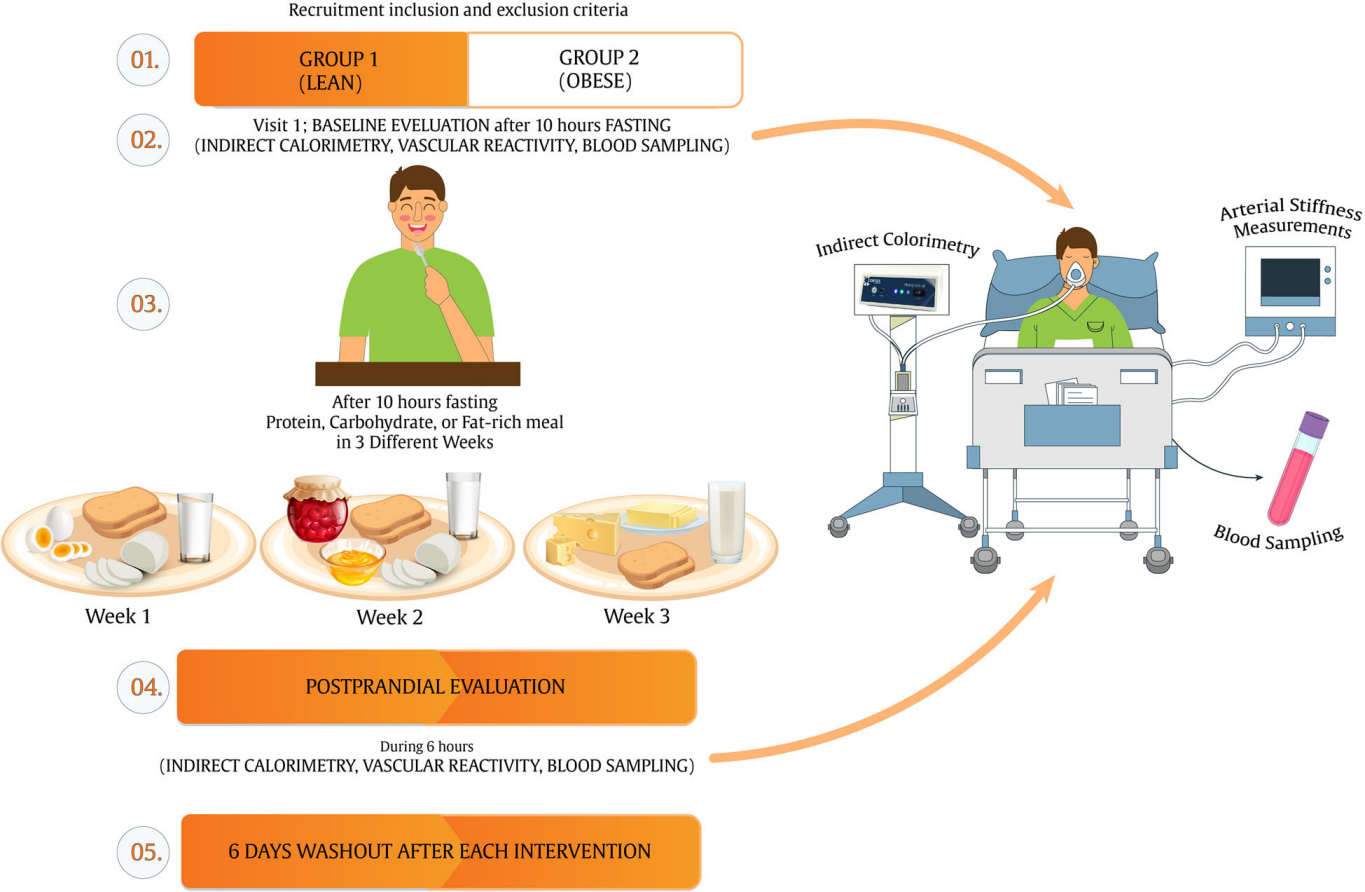
Study diagram of enrollment, interventions, and assessments

The study protocol is based on the SPIRIT guidelines [30]. Figure 1 depicts the diagram of enrollment, intervention, and assessments. Table 1 shows the schedule of enrollment, intervention, and data collection for each participant. This is a parallel clinical trial, and the subjects will consume three different test meals (high-carbohydrate, high-fat, and high-protein) on different days with a 1-week interval between each intervention.

**Table 1:** SPIRIT 2013 figure showing schedule of enrolment, intervention, and data collection for each participant. ^a^Randomly assigned meal

Timepoint	Study period					
	Enrollment	Allocation	Post-allocation			
	Preintervention	Time 0	Baseline	Intervention visit 1	Intervention visit 2	Intervention visit 3
**Enrolment** Eligibility screen Informed consent Allocation	✓					
	✓					
		✓				
**Interventions** High protein High charbohydrate High fat				✓^a^	✓^a^	✓^a^
				✓^a^	✓^a^	✓^a^
				✓^a^	✓^a^	✓^a^
**Data collection** Food frequency Questionnaire		✓				
International physical activity questionnaires		✓				
Anthropometric variable		✓				
FAT and Lean mass		✓				
Energy expenditure			✓	✓	✓	✓
Resting metabolic rate			✓	✓	✓	✓
Charbohydrate oxidation			✓	✓	✓	✓
Fat oxidation			✓	✓	✓	✓
Protein oxidation			✓	✓	✓	✓
Blood pressure			✓	✓	✓	✓
Heart rate			✓	✓	✓	✓
Augmentation index			✓	✓	✓	✓
Pulse wave velocity			✓	✓	✓	✓
Serum glucose and insulin levels			✓	✓	✓	✓
Lipid profile			✓	✓	✓	✓

### Participants and data collection

Data collection will be performed by two nutritionists and one nurse, who have been trained on the proper use of various devices and are able to complete the questionnaires accurately. Since this is a pilot study, we cannot assess the attrition rate, and it is also not necessary to define and calculate the sample size. Our study follows sample size for pilot trial and aims to have at least 12 subjects per group who provide full data( in total 24 subjects) [31]. This sample size will to provide a pooled standard deviation that provides enough precision for the development of a future study.

### Study setting and sample population

This pilot, a parallel clinical trial will be performed at Imam Reza Hospital of Mashhad, located in the northeast of Iran. The participants will be recruited by local advertising with posters distributed at Mashhad University of Medical Sciences.

### Inclusion criteria

The inclusion criteria of the study are as follows: (1) age of 18–40 years; (2) adults with normal weight (body mass index [BMI]: 18.5–23.5 kg/m^2^, body fat percentage: 12–22%, waist circumference < 90 cm), and obese adults (BMI > 27.5 kg/m^2^, body fat percentage > 27%, waist circumference > 95 cm); (3) apparently healthy men; (4) written informed consent to participate; and (5) willingness to undertake the required fasting periods.

### Exclusion criteria

The exclusion criteria of the study are as follows: (1) professional athletes; (2) greater changes in the bodyweight than 10% within the past 6 months; (3) current smoking habits; (4) use of medications or supplements affecting the metabolism (e.g., thyroid drugs, caffeine); (5) history of CVDs, hypertension, diabetes mellitus, hyperlipidemia, and neurological and/or neuropsychological disorders; (6) consumption of toxic substances; (7) use of supplements for weight loss or weight gain; and (8) inability to partake in the intervention due to intolerance/dietary preferences.

### Test meals

Three interventions will be implemented in the current research, involving the consumption of high-fat, high-carbohydrate, and high-protein meals as the test meals in a randomized order. On the day of the intervention, the TEE of the subjects will be determined via indirect calorimetry. The test meals will contain 25% TEE for men, and the high-protein meal will consist of bread, cheese, boiled eggs, and skimmed milk (30% protein, 50% carbohydrates, and 20% fat). The high-carbohydrate meal will consist of skimmed milk, white bread, butter, jam, and honey (70% carbohydrates, 20% fat, and 10% protein), and the high-fat meal will contain bread, butter, cream cheese, high-fat milk, and jam (50% fat, 10% protein, and 40% carbohydrates). The subjects will be asked to ingest the test meals within a maximum of 15 min on each test day.

#### Outcomes

The primary outcome will to evaluate the feasibility of conducting a trial to compare the effect of three test meals in lean and obese men. The feasibility will be assessed by measuring subject compliance with the intake of three test meals, recruitment, and retention rate in 3 days of study. Also, adherence to sample collection protocol will be assessed. Secondary outcomes will to compare the effect of three test meals on metabolic responses and vascular parameters in lean and obese men for sample size calculation.

#### Clinical outcomes

Clinical outcomes will be the following: (1) metabolic responses, will be evaluated by metalyzer device and blood sampling and (2) arterial stiffness, will be evaluated by sphygmocor device.

#### Feasibility outcomes

The feasibility of recruitment will be determined by calculating the proportion of enrolled participants from those screened for eligibility from the varied recruitment sources. Retention will be measured by the percentage of participants completing the study through the post-assessment. Intervention adherence will be measured from the information recorded by a nutritionist at three test days. The study will be deemed successful if (1) 60% recruitment target is achieved, (2) 80% or more of the subjects completed this study, and (3) subjects and investigators report acceptability of the study and adherence to the sample collection protocol will be reviewed by investigators.

## Statistical analysis

Briefly, descriptive statistics will be used to summarize enrollment adherence, recruitment rates, and dropout. Feasibility outcomes will be presented as percentage and 95% confidence intervals. A retention rate of > 80% will be considered as feasible to conduct a future RCT. Analysis of outcomes will be conducted using both intention to treat. Also, in the analysis of clinical outcomes, descriptive statistics will be used to compare and determine the primary characteristics of the study groups, and the normal distribution of the variables will be evaluated using the Kolmogorov-Smirnov test. In total, 24 subjects have been assigned to the obese and normal groups. The baseline comparisons between the groups will be performed using the independent samples *t* test or Mann-Whitney *U* as appropriate. In addition, repeated-measures analysis of variance (ANOVA) will be used to assess the timing effect of the research parameters in various phases of the study. Data will be used for sample size estimation and to determine the most appropriate outcome measure for the main study.

### Measurement tools

#### Anthropometric parameters and body composition

The anthropometric measurements will be performed at the outset of the fasting state by a trained nutritionist. In addition, bodyweight will be measured to the nearest 0.1 kg, with the participants in light clothing. The height of the subjects will be measured using a stadiometer in the standing position to the nearest 0.1 cm. BMI will be defined as the weight (kg) divided by the square of height (m^2^), and waist circumference will be measured at the midline between the iliac crest and lowest ribs to the nearest 0.5 cm. Finally, the body composition of the participants will be determined using bioelectrical impedance analysis (AVIS 333).

#### Dietary measurements

Dietary intakes will be assessed using the valid and reliable food frequency questionnaire [32], and the collected data will be expressed as gram per day using household measures. In addition, the modified food consumption database of the US Department of Agriculture will be used to calculate the daily nutrient intake of each subject [33].

#### Physical activity

The level of physical activity in the participant will be evaluated using a validated questionnaire [34] and calculated based on the metabolic equivalent tasks. Based on the questionnaire, the participants will be divided into three groups of low, medium, and high physical activity.

#### Screening questionnaires

A screening questionnaire will be designed based on a researcher-made questionnaire and the available standardized questionnaires in order to acquire the general data of the participants. Accordingly, the subjects will be excluded for medical reasons such as hypertension, CVDs, hypothyroidism/hyperthyroidism, intolerance, allergies, diabetes mellitus, and other acute/chronic diseases.

#### Indirect calorimetry

At this stage, the participants will be asked to stay awake and motionless in the supine position, and air samples will be collected using a mask (MetaLyzer 3B-R3 device). Moreover, respiratory gas exchange measurements will be recorded for 20 min in the fasting state, followed by hourly measurements for 6 h after the consumption of the test meals. Furthermore, fasting REE will be measured in a quiet area at room temperature, with the participants in the supine position. The respiratory quotient will also be calculated based on the oxygen consumption and carbon dioxide production, and TEF will be measured as the difference of the postprandial subtracted by the fasting REE. The resting energy expenditure and substrate utilization will also be estimated via indirect calorimetry after resting for 30 min.

#### Pulse wave analysis

All the patients will receive an ultrasound examination of the carotid artery in the supine position, with the head turned to 45° from the side to be scanned and the operator placed on their right side. In addition, brachial blood pressure evaluation and pulse wave analysis will be conducted in the supine position using the Sphygmocor XCEL device. Blood pressure will be measured after the minimum rest of 15 min on the right upper arm in a quiet area. The augmentation index (AIx), central blood pressure (cBP), and heart rate (HR) will also be analyzed in accordance with the guidelines of the pulse wave analysis device manufacturer. After the measurement of the HR, cBP, and AIx will be estimated using built-in algorithms.

#### Carotid-radial PWV

Carotid-radial pulse wave velocity (PWV_b_) is a measure of arterial stiffness, which will be determined based on the sequentially measured electrocardiogram-gated left carotid and radial waveforms (applanation tonometry) using the foot-to-foot method to determine the pulse travel time in our study. Moreover, the travel distance of the pulse wave will be calculated as the difference in the distance between the suprasternal notch and each recording site using a tape measure over the body surface. The measurements will be performed at least twice, and the mean PWV_b_ will be applied to the analysis. PWV_b_ will be assessed at baseline and 30, 90, 150, 210, 270, and 330 min after the test meals.

#### Blood samples

Each test day will be implemented at 7 AM–2 PM; this period was selected since it was assumed to represent a common interval between breakfast and lunch. Serum samples will be collected before meal consumption and 60, 180, and 360 min after the meal initiation to evaluate metabolic activity markers. A maximum of 3 ml of the blood will be collected from each patient at each of the given times (12 ml/day) for the analysis of insulin, glucose, FFAs, TG, low-density lipoprotein, high-density lipoprotein, and total cholesterol. The blood samples will be collected at room temperature and immediately centrifuged, and the serum samples will be frozen at the temperature of −20 °C.

#### Experimental protocol

The day before each of the three test days, the subjects will prepare their own meals and a standardized daily menu plan, which will consist of 15% protein, 55% carbohydrates, and 30% fat based on their daily energy requirements. The diets will ensure equally filled glycogen stores and similar macronutrient balance in each subject on every test day. The subjects will not be allowed to engage in severe physical activity within 2 days prior to the test days. On the test day, the subjects will arrive at the research center at Imam Reza Hospital at 7 AM with minimum activity (by car/bus) and after fasting from food, drinks (except water), caffeine, and alcohol for the past 12 h.

**Fig. 2:**
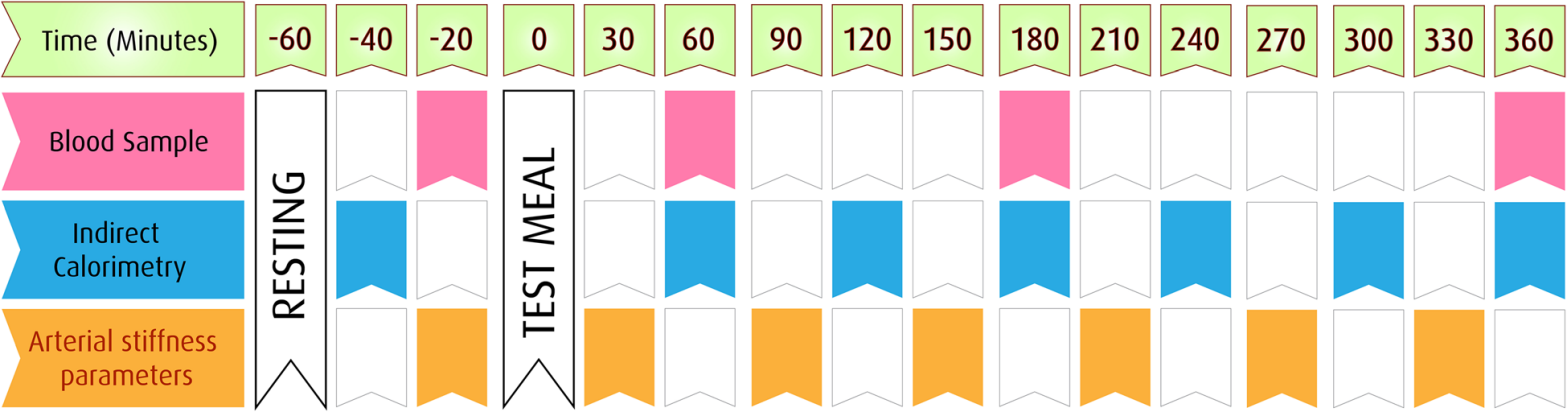
Scheduling study days

Before the tests, all the subjects will rest in the supine position for 30 min, lying on a bed in the semirecumbent position until the end of the tests. Initially, all the measurements will be performed in the fasting state and postprandially within 6 h. At the next stage, the participants will randomly receive the test meals and given 15 min to take the meals under the supervision of the researcher until the test meal is completely consumed. Afterwards, the participants will complete a series of tests, including indirect calorimetry, PWV, and pulse wave analysis. In addition, blood samples will be collected within 6 h. All the tests will be carried out in the exact same conditions (e.g., temperature-regulated room and quiet area). Following the test meals, the participants will fast again, refraining from food and drinks (except water) for another 6 h while the testing stage will continue. The testing conditions will be repeated on the next two test days. Figure 2 depicts the schedule of the study days.

## Discussion

The current research aims to investigate the effects of the single ingestion of various test meals on the metabolic responses and vascular reactivity of healthy and obese adults. To the best of our knowledge, no previous research has concurrently assessed the postprandial effects of three meals with various compositions (high-protein, high-fat, and high-carbohydrate) on metabolic and vascular parameters. Based on the study design, the subjects will receive three types of meal on different test days to determine the differences in the metabolic parameters and arterial stiffness indices between the normal and obese subjects.

Considering the design of the present study, the balance in the confounding variables will be ensured in the study groups, and the bias induced by the characteristics of the subjects will be removed. However, the main challenge in this experiment will be the attrition rate and subject compliance as they must attend the study setting on three days, and the study process will continue for almost 7 h for each subject on each test day. Consequently, some of the subjects may fail to complete the three test days due to lengthiness, tardiness, and other reasons. Notably, the researchers will thoroughly describe the principles and requirements to the subjects prior to the experiment in order to ensure their compliance.

Conflicting results have been proposed regarding the effects of meal composition on metabolic and vascular responses. Based on the hypothesis of the current research, our findings will describe the effects of meal composition on metabolic and vascular responses. Given the increasing prevalence of CVDs and their close correlation with dietary habits, lipemia, and glycemia after meals, investigating the effects of meal composition on vascular functions and metabolic responses is of great interest. Furthermore, the subject matter may be incorporated into the design of novel lifestyle and dietary interventions. We recognize that in this pilot study, we are not powered to investigate effects between the different groups. Collecting data on the secondary outcomes such as metabolic and vascular responses will help to inform sample size calculations in future studies.

## Conclusion

If a significant result is achieved regarding the measured parameters in the pilot study, we will design a study with a larger sample size consisting of both genders. The results of these studies may provide the proper tools to adapt various meal types to different body compositions in order to improve metabolic responses and vascular reactivity.

## Data Availability

N/A.

## Abbreviations

PVW: Pulse wave velocity
BMI: Body mass index
FFA: Free fatty acid
CVD: Cardiovascular disease
TG: Triglyceride
